# Sterile water injections for relief of labour pain (the SATURN trial): study protocol for a randomised controlled trial

**DOI:** 10.1186/s13063-022-06093-3

**Published:** 2022-02-16

**Authors:** Nigel Lee, Yu Gao, Lena B. Mårtensson, Leonie Callaway, Belinda Barnett, Sue Kildea

**Affiliations:** 1 School of Nursing Midwifery and Social Work, University of Queensland, Level 3 Chamberlain Building, St Lucia, Queensland Australia; 2 Molly Wardaguga Research Centre, College of Nursing & Midwifery, Charles Darwin University, Level 11, East building, 410 Ann St, Brisbane, Queensland 4000 Australia; 3 School of Health Sciences, University of Skovde, Box 408, SE541 28 Skövde, Sweden; 4 Women’s and Newborn Services, Royal Brisbane and Women’s Hospital, Level 6, Ned Hanlon Building, Butterfield Street, Herston, Queensland 4029 Australia; 5 Molly Wardaguga Research Centre, College of Nursing & Midwifery, Charles Darwin University, 17 Grevillea Drive, Sadadeen, Alice Springs, 0870 Australia

**Keywords:** Sterile water injections, Labour pain, Analgesia, Randomised controlled trial

## Abstract

**Background:**

Up to 80% of women use some form of pharmacological analgesia during labour and birth. The side effects of pharmacological agents are often incompatible with the concurrent use of non-pharmacological pain relieving strategies, such as water immersion, ambulation and upright positioning, or may have negative effects on both the mother and fetus. Sterile water injections given into the skin of the lumbar region have been demonstrated to reduce back pain during labour. However, the injections given for back pain have no effect on abdominal contraction pain. The analgesic efficacy of sterile water injections for abdominal pain during childbirth is unknown. The injections cause an immediate, brief but significant pain that deters some women from using the procedure. This study aims to investigate the use of water injections given intradermally into the abdomen to relieve labour contraction pain. A vapocoolant spray will be applied to the skin immediately prior to the injections to reduce the injection pain.

**Methods:**

In this pragmatic, placebo controlled trial 154 low-risk women in labour at term with a labour pain score ≥ 60 on a 100 millimitre visual analogue scale (VAS) will be randomly allocated to receive either six injections of sterile water or a sodium chloride 0.9% solution as a placebo (0.1–.0.3 ml per injection). Three injections are given along the midline from the fundus to the supra-pubis and three laterally across the supra-pubis. The primary outcome will be the difference in VAS score 30 min post injection between groups. Secondary outcomes include VAS score of the injection pain on administration, VAS score of labour pain at 60 and 90 min, maternal and neonatal birth outcomes.

**Discussion:**

Access to effective pain relief during labour is fundamental to respectful and safe maternity care. Pharmacological analgesics should support rather than limit other non-pharmacological strategies. Sterile water injections have the potential to provide an alternative form of labour pain relief that is easy to administer in any labour and birth setting, and compatible with other non-pharmacological choices.

**Trial registration:**

ANZCTR (ACTRN12621001036808) Date submitted: 22/06/2021. Date registered: 05/08/2021. https://www.anzctr.org.au/

**Supplementary Information:**

The online version contains supplementary material available at 10.1186/s13063-022-06093-3.

## Administrative information

**Table:** 

Title	Sterile water injections for relief of labour pain (the SATURN trial): Study protocol for a randomised controlled trial
Trial registration	anzctr.org.au (ACTRN12621001036808) Date submitted: 22/06/2021. Date registered: 05/08/2021.
Protocol version	Version 3 22 November 2021
Funding	The trial was funded by the Australian Government Department of Health Medical Research Future Fund (APP2006488)
Author details	^1^ School of Nursing Midwifery and Social Work University of Queensland St Lucia, Queensland Australia 4072 ^2^ Molly Wardaguga Research Centre College of Nursing & Midwifery Charles Darwin University Level 11, East building, 410 Ann St, Brisbane Queensland. Australia 4000 ^3^ School of Health Sciences University of Skovde, Box 408, Sweden SE541 28 ^4^ Women’s and Newborn Services, Level 6, Ned Hanlon Building Royal Brisbane and Women’s Hospital Butterfield Street, Herston Queensland, Australia. 4059
Name and contact information for the trial sponsor {5b}	Dr Nigel Lee School of Nursing Midwifery and Social Work University of Queensland St Lucia, Queensland Australia 4072 nigel.lee@uq.edu.au
Role of sponsor {5c}	The University of Queensland is the trial sponsor. Final decisions related to the study design, data collection, analysis, interpretation, and manuscript preparation were made by the investigator-team.

## Background

In countries such as Australia and the United Kingdom up to 80% of laboring women use some form of pharmacological analgesia [1, 2]. Current options for pharmacological analgesia in labour have changed little in past decades with the most common choices being opioids, nitrous oxide inhalation and neuraxial (epidural) analgesia. The analgesic effectiveness of opioids such as morphine and pethidine is minimal with most women continuing to report moderate to severe pain [3]. Opioids are more likely to provide drowsiness than analgesia which has highlighted the ethical problem of primarily providing sedation in response to a woman’s request for pain relief [4]. Opioids readily cross the placenta and are found in breast milk.. The metabolism of pethidine results in the formation of norpethidine, which is associated with neuronal depression in the neonate up to 60 h post-birth and feeding difficulties for up to 6 weeks post partum [5, 6].

The analgesic effectiveness of nitrous oxide varies from no difference to the placebo to similar to that opioids [7]. Whilst generally considered safe recent studies have highlighted metabolic, oxidative, genotoxic, and transgenerational epigenetic effects from prolonged exposure. A 1 to 3 h exposure to 50% nitrous oxide (a common dose during labour) inactivates methionine synthase in the mother and fetus which can take 3 to 5 days to recover. This increases the potential for haematological disorders such hypercoagulation, particularly in Vitamin B12 deficient women [8].

In high income countries epidurals are used by up to 70% of labouring women [1, 9]. Epidurals have been shown to provide more effective analgesia than opioids [10]. Whilst generally considered safe epidurals can have immediate, medium and possibly long term side effects. Epidurals are strongly associated with maternal fever during labour resulting in increased use of antibiotics [11], prolonged labour and assisted birth (vacuum extraction or forceps) [12].

Many women will use a combination of pharmacological and non-pharmacological strategies to achieve personal and psychological control over the pain they are experiencing, rather than seeking a total elimination of pain [13]. All of the current pharmacological agents are largely incompatible with non-pharmacological options, particularly those involving ambulation, upright positions or water immersion [14]. Both nitrous oxide and opioids can result in sedation and impaired balance that may increase the risk of falls injuries. Epidurals require intravenous cannulation, fluid administration, urinary catheterisation and continuous fetal monitoring. These restrict mobility and reduce a woman’s ability to adopt favourable positions for labour and birth.

A recent placebo controlled trial demonstrated the efficacy of sterile water injections into the lumbar region to relieve back pain in labour with no detrimental side effects [15]. Back pain in labour is different to and may occur independently from abdominal labour pain [16]. Furthermore, the injections given into the lumbar region for back pain have no effect on abdominal contraction pain [15, 17]. Injections of sterile water are acutely painful for a brief period and this is known to act as a deterrent to both women and clinicians [18, 19]. Theoretically, the acute pain associated with the injection (noxious stimulus), tissue distension and increased osmotic pressure stimulate gate control of pain and endorphin release to reduce pain [20, 21]. A moderate reduction in injection pain, through the administration of one rather than four injections, still results in significant analgesia though for a shorter duration [17]. Though no reliable method to achieve this is currently in use [17]. A non-placebo exploratory trial and case study suggests the potential for using water injections to relieve abdominal labour pain [22, 23].

Vapocoolant sprays consist of rapidly evaporating solvents that quickly reduce skin temperature to produce a numbing effect and result in a moderate reduction in pain scores [24]. The only reported side effects are the cold sensation and occasionally mild transient erythema at the treated sites [24].

## Objectives

We aim to test the effectiveness of sterile water injections given into the abdomen to relieve labour contraction pain. We will also apply a vapocoolant spray immediately prior to the injections to reduce the injection discomfort.

## Methods: participants, interventions and outcomes

### Trial design

**Fig. 1:**
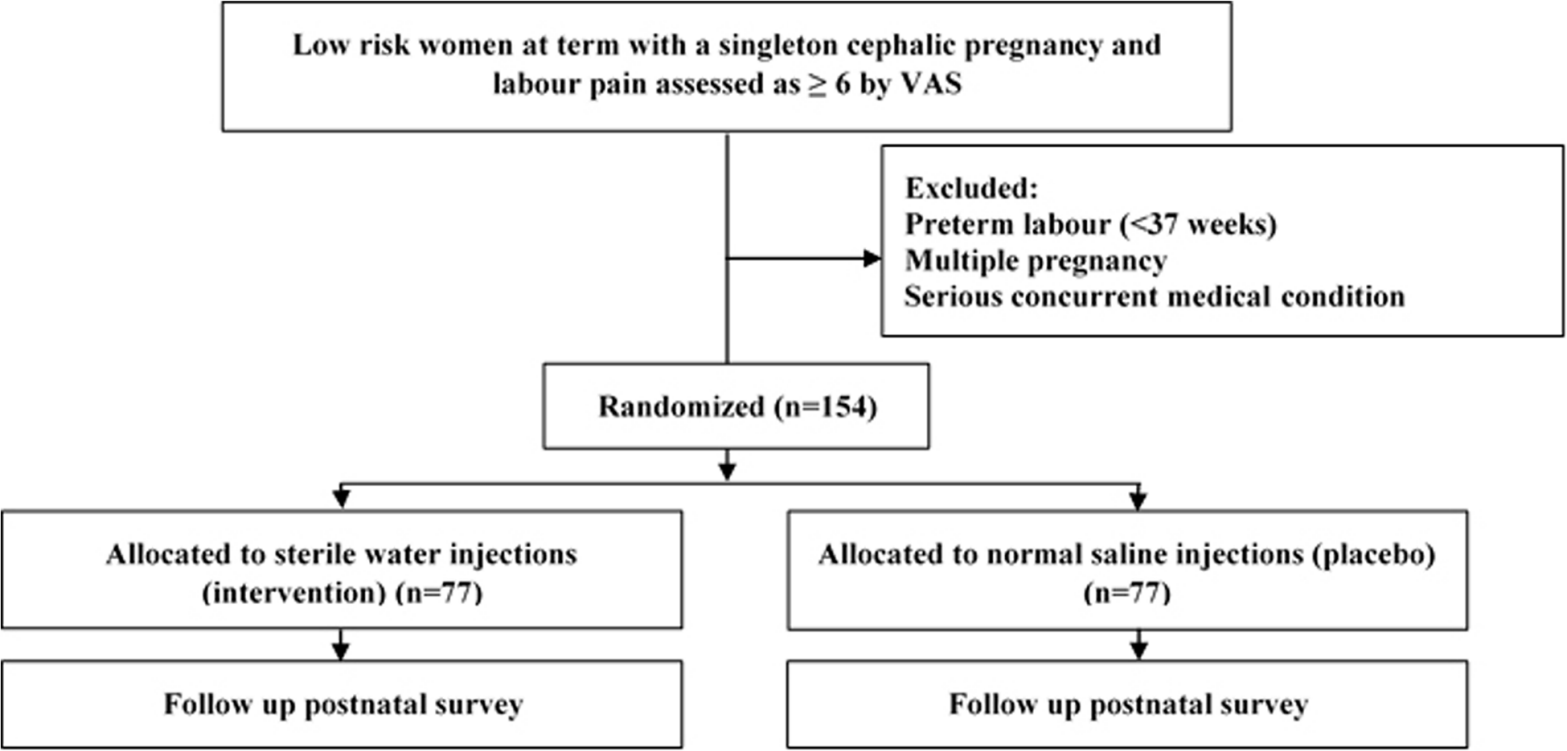
Participant flow

This will be a pragmatic randomised placebo-controlled superiority trial specifically designed to provide evidence of the efficacy of water injections to relieve abdominal labour pain. Following provision of informed consent participants will be randomised to receive either water injections (intervention) or normal saline injections (control). For both groups pre-injection preparation with a vapocoolant spray will occur. The participant flow is demonstrated in Fig. 1.

### Study setting

The trial will be conducted at a tertiary maternity unit in Brisbane Australia providing labour and birth care for approximately 5000 women annually. The research site provides both birth-centre and standard labour and birth care.

### Study population and recruitment

We plan to recruit and consent provisionally eligible women in the late antenatal period (36–37 weeks). Whilst this may include women who are either ineligible at the onset of labour, or remain eligible but decline participation, this strategy will reduce the need to rely on clinical staff, in the birth suite and birth centre, to identify, screen, provide participant information and complete consent documentation whilst also providing care. This also minimises the ethical issue of recruiting and consenting in labour when pain and/or effect of medications may impact on the woman’s capacity to consent to participation in research. Women may also be recruited upon presentation to the Birth Centre or Birth Suite in early labour.

The study site offers a number of models providing care to women likely to meet the inclusion criteria. These are broadly defined as Midwifery and General Practitioner (GP) Shared Care. Midwifery care models are provided by the Birth Centre, Midwifery Group Practices (MGP). Women attending GP shared care all return at 36 weeks gestation, providing an opportunity to discuss the trial and participation. Women attending Birth Centre and MGP antenatal clinics will be approached during their 36–37 week visit. Consent processes will be undertaken by the Research Midwives or in their absence, a midwife who is not providing direct care to the woman.

In the event that COVID-19 restrictions prevent a face to face consent process Potential participants will be provisionally approached during the routine antenatal video conference and asked if they are interested in discussing participation in the trial with a research midwife. Interested participants will be asked for contact details (email address, mobile phone number) to arrange a suitable time for a discussion regarding the trial and potential participation using their preferred video conferencing format (e.g. FaceTime, Zoom, Teams). A link to the SATURN Trial Participant Information and e-Consent form will be sent to the potential participant so they can review the trial information and consider any questions prior to the discussion.

The e-Consent form requires a three step verification process consisting of i) indicating ‘yes’ to verify statements that appear on the consent form; ii) drawing a signature on the screen either with a mouse or device touch screen; iii) final declaration that the participant is aware that submitting the form with the signature is equivalent to providing a signed consent form. The participants are then able to download a copy of the completed and signed SATURN trial Participant Information and e-Consent form and a pdf copy is sent to a designated email address.

This trial does not involve the collection of biological specimens.

### Eligibility criteria

Eligibility criteria consists of ≥16 years of age, singleton cephalic (head down) pregnancy, ≥ 37 weeks gestation, spontaneous or induced labour, no serious concurrent medical conditions (pre-eclampsia, coagulopathy, diabetes other than diet controlled), cognitively capable of providing consent, able to read and understand instructions written in English. Upon admission to the birth suite randomisation will occur when the participant’s self-assessment of labour pain ≥60 mm (Visual Analogue Scale [VAS] 0-100 mm, 0 = no pain 100 = worst conceivable pain), and requesting pain relief.

### Randomisation and blinding

Randomisation schedules will be prepared by a statistician independent of the study using computer-generated pseudo-random numbers, using varying block sizes. Identical ampoules of either normal saline or sterile water will be pre-prepared by the study site pharmacy and packed in opaque black plastic packets and arranged based on the allocation schedule. Following confirmation of consent two midwives will remove the next ampoule in sequence and administer the injections. Normal saline for injection 0.9% will be used as the placebo solution. Normal saline is an active placebo resulting in some minor injection discomfort and some degree of analgesia, it has been used successfully in previous placebo controlled trials [15, 25]. Outcome assessment will be undertaking by the midwife administering the injections. The use of the vapocoolant in both the intervention and control arms will reduce the chance of participant and outcome assessor unbinding as all women will experience cooling effect of the vapocoolant. The data analysis will be blinded to the allocated group.

### Interventions

**Fig. 2:**
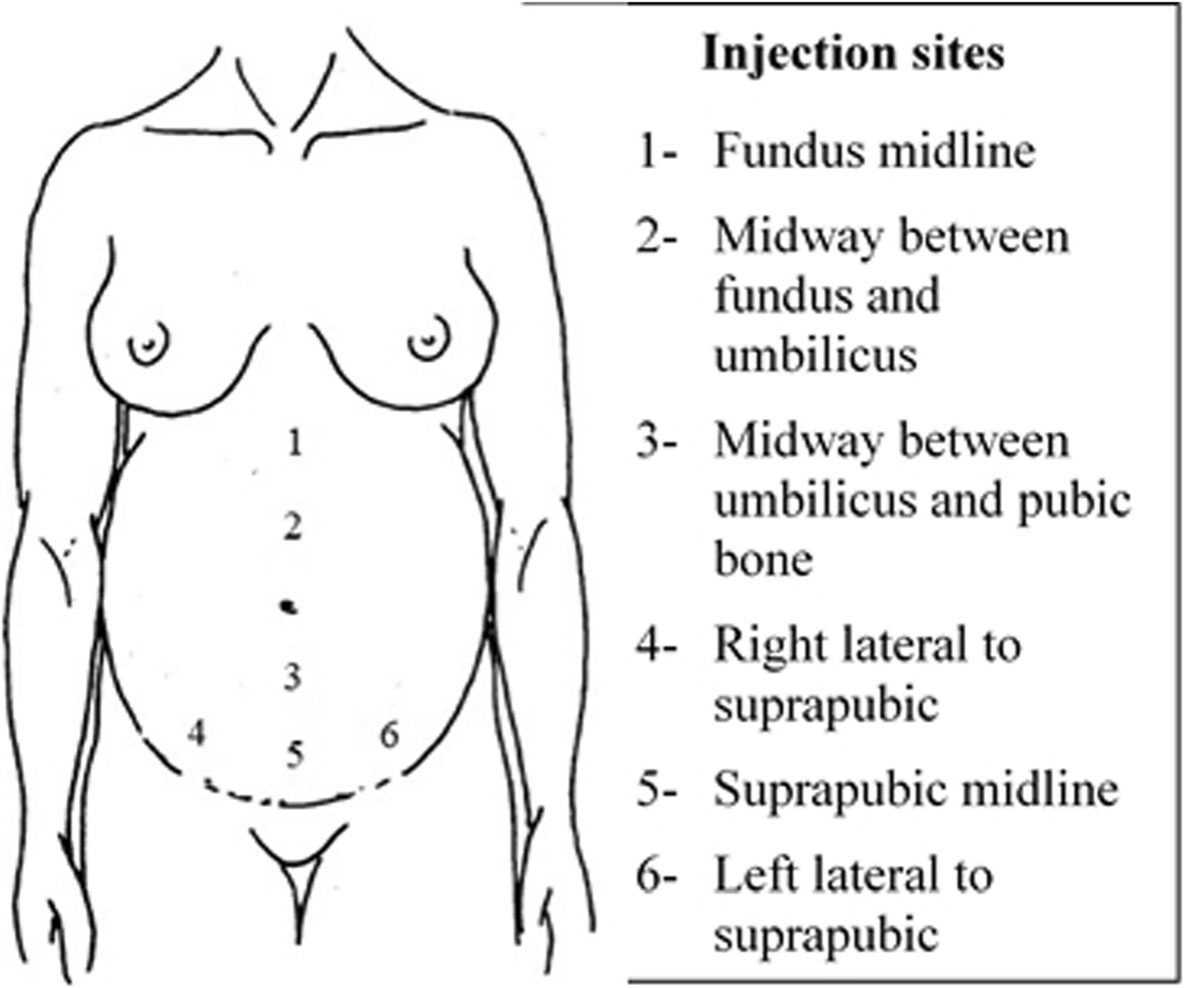
Injection sites

Participants in the intervention groups will receive intradermal injections of 0.1–0.3 ml of sterile water into six anatomical points on the abdomen (Fig. 2). The volume required to be injected is based on the visual estimation of the resulting blister or ‘bleb’. If the needle is inserted beyond the intradermal layer the bleb does not occur and the needle may require repositioning to achieve the correct anatomical depth. The location of the injections is based on a previous RCT of water injections versus acupuncture and early studies that used intradermal injections of local anaesthetic into the abdomen to relieve pain in labour [22, 26, 27]. These studies suggested that injections given in a line extending medially from the fundus to the suprapubic area and extending laterally from the suprapubic region will produce an effective analgesic response. As the experience and location of contraction pain varies considerably the precise location and number of injections may vary based on the areas of greatest discomfort indicated by the woman. Otherwise there will be no special criteria for modifying the allocated interventions. The injection procedure will be ceased at the request of the participant. Up to three repeat courses of injections will be provided for women who request them.

Data items such as location of injections, VAS and duration of effect will be collected for the initial and repeat injections. To improve adherence to study protocols all midwives administering water injections will be credentialed in this technique using a competency based assessment that was used successfully in our previous trials [22, 28]. Midwives will indicate the number and location of the injections given on the data collection form which will be monitored by the research officers for adherence to the protocol.

Participants in the control group will receive intradermal injections of 0.1–0.3 ml of normal saline 0.9% for injection into the abdomen as previously described. All other components of the administration will be the same as the intervention group.

Both groups will also receive skin preparation immediately prior to the injections using the vapocoolant spray PainEase® manufactured by Gebauer. PainEase® spray contains 1,1,1,3,3-Pentafluoropropane and 1,1,1,2-Tetrafluoroethane which are non-flammable and unlike ethyl chloride are not absorbed through the skin and therefore safe to use in pregnancy. The cooling effect occurs through the rapid evaporation. PainEase is registered with the Australian Therapeutic Goods Administration as a vapocoolant topical anaesthetic available to the general public. The injection points will be sprayed with PainEase® spray at a distance of 12 cms from skin for a period of 5 to 8 s immediately prior to administering the injections*.*

Implementing the injections of sterile water of saline will not require alteration to usual care pathways. Participants in both arms will have access to any form of standard care including combinations of currently available pharmacological and non-pharmacological options.

Provisions for post-trial care There is no anticipated harm and compensation for trial participation. In the event that participants experience harm as a result of participation they will be able to apply for compensation through trial insurance provided by the sponsor.

### Outcomes

The primary outcome for the trial is the mean difference in VAS scores of labour pain between intervention and control groups at 30 min following the administration of the injections. Secondary outcomes include the number of women experiencing an at least 30% or 50% reduction in pain following the injections. This is the measure of analgesic effectiveness recommended in the Cochrane Review of water injections for back pain in labour [29]. Other secondary outcomes are detailed in Table 1.

**Table 1:** Schedule of enrolment, interventions and assessments

		Study period					
			Post-allocation				Close-out
	Enrolment	Allocation	*Time periods after injection*			*1–14 days post birth*	*From perinatal database*
TIMEPOINT**			*30 min*	*60 min*	*90 min*		
**ENROLMENT:**							
**Eligibility screening**	X	X					
**Signed consent form**	X						
**Randomisation**		X					
**INTERVENTIONS:**							
***Sterile water injection***		X					
***Normal Saline placebo injection***		X					
***Vapocoolant spray***		X					
**ASSESSMENTS:**							
***Cervical dilation***		X					
***VAS of labour pain prior to injections***		X					
***VAS of injection pain***		X					
***VAS of labour pain post injection***			X	X	X		
***At least 30% reduction in VAS of pain***			X				
***At least 50% reduction in VAS of pain***			X				
***Pharmacological analgesia use***		X					X
***Non-pharmacological analgesia use***		X					X
***Duration of labour***							X
***Augmentation of labour***							X
***Mode of birth***							X
***Estimated blood loss at birth***							X
***Apgar scores***							X
***Type of neonatal resuscitation***							X
***Admission to nursery***							X
***Duration of hospital stay***							X
***Breastfeeding at discharge***							X
***Postnatal survey***						X	
***Economic analysis***							X

### Postnatal survey

Following birth all participating women will be asked to complete an electronic survey of their experiences of the injections and spray they received in the context of their labour and birth.

The survey is based on versions used in our previous studies and measures levels of satisfaction with pain relief, relaxation, likelihood to use in a subsequent labour and recommend to other women, most positive and negative aspects of the trial experience [15]. Survey data will be identifiable only through the allocated study participant code to enable matching with the allocated group in the clinical trial.

### Participant retention and completion of follow-up

As the primary and clinical outcomes data is collected prior to or at birth loss to follow-up is expected to be minimal. Therefore, there are no specific plans to promote participant retention beyond this timeframe. Reasons for discontinuing the injections or declining further rounds of injections (i.e. birth, progress to use of epidural, other maternal reason, other fetal reason (e.g. abnormal CTG)) will be collected by the midwife on the data collection form. For women who remain in hospital following their birth the survey will be completed on a tablet device. For those women opting for early discharge or discharged on weekends, a link to the survey will be emailed with one follow up contact from a research assistant within 2 weeks.

### Sample size

We have calculated the sample size based on the recommended minimal clinically significant reduction in VAS scores: 10 mm difference on a 100 mm VAS score between intervention and placebo [30]. To demonstrate a 10 mm reduction (SD 20 mm) in VAS scores with 80% power and 0.05 significance (two-sided) would require 64 participants per group. Based on previous studies [15] we estimate an attrition of 20% due primarily to women giving birth or requesting epidural analgesia prior to the measurement of the primary outcome. The total sample size required would be 154 participants, 77 per trial group.

### COVID-19 safe data collection

We have designed a method of data collection that is both COVID-19 safe and compatible with the realities of providing care during labour and birth. Data will be entered directly into a REDCap database using an iPad specifically configured with the REDCap mobile application for the trial. Participants will carry a card that contains a unique study number that once entered will link to an existing database entry. Following randomisation the attending midwife will enter minimal required data (e.g. allocation code, results of most recent vaginal examination). The iPad will then be placed in a waterproof protective sleeve that will allow the woman to use touch screen ‘sliders’ to indicate their level of pre–treatment, injection and post-treatment on a 100 mm VAS scale. The use of digital VAS has been validated against paper versions [31]. The protective sleeve will prevent device contamination from body fluids, shower and bath water and can be disposed of following data entry. Direct data entry will reduce data transcription errors contributing to trial fidelity. Each midwife will have a unique code to identify them as responsible for data entry in keeping with Good Clinical Practice (GCP) principles. A horizontal VAS is used to measure the primary outcome, the experience of self-reported pain scores. The VAS is sensitive to pain intensity, validated for use in research and most individuals have no difficulties using it [32]. Demographic and clinical data will be extracted from the research sites perinatal database.

### Data management

Data entry, both paper and electronic, will be stored and maintained at the University of Queensland as specified by the relevant UQ Policies and Procedures, specifically: 4.20.06 Research Data Management, 6.40.01 Information Management Policy. Paper files will be stored within a locked cabinet with a locked office with only relevant research team members provided with access. Electronic files will be stored on a secure password protected network with access restricted to relevant research team members. Data sharing arrangements will specify transfer of required data using secure password protected files and file transfer platforms or restricted access to cloud based datasets (REDcap). The final dataset will only be accessible to investigators participating in the data analysis as specified within the trial agreements.

### Data analysis

All women who underwent randomisation and for whom primary outcome data is available will be analysed in their allocated treatment groups regardless of the intervention received (i.e. intention to treat). Randomisation should ensure that any baseline differences between groups occur by chance. To control for cluster effect repeated measurement, a linear mixed-model analysis will be conducted to investigate the difference in mean VAS score pre and post intervention. Missing VAS data will be addressed through imputation (last observation carried forward) A sensitivity analysis will be conducted following imputation to assess any effect on findings. Clinical data will be analysed on an available case basis with numbers of participants with missing data presented in the results tables. Categorical data will be analysed with chi-squared tests and non-repeated continuous variables will be analysed with t-tests if normally distributed or Mann-Whitney U test if non-normally distributed. Additional multivariable analysis will be employed if baseline differences are noted between the two groups. Treatment effects will be presented as mean difference or relative risks with 95% confidence intervals. All study outcomes will be analysed using a two-sided *P* value of < 0.05 to indicate statistical significance. We do not plan to undertake any subgroup analysis or interim analysis.

For the postnatal survey data descriptive statistics will be calculated for all variables, including mean, median, standard deviation, range, and percentages as appropriate. Free text responses will be analysed thematically.

### Economic analysis

A cost-effectiveness analysis will be undertaken to compare costs and outcomes of between intervention and placebo groups from the perspective of the health system. The approach to identification, measurement and valuing resource use will follow a similar approach to that which we have used in previous studies [15]. Information about resource use and costs will be collected at the research site. In the case of the intervention, resources to be identified and measured will include those associated with the establishment of the intervention (staff training, development of educational resources and credentialing) and staff time required for administration. Costs will be allocated to the relevant resource items using appropriate values. For example, staff time will be costed using hourly award rates plus on-costs; and the costs of length of stay and complications from admission to discharge of hospital will be estimated using appropriate Diagnostic Related Groups. The outcome for the economic evaluation is VAS pain score difference between two groups. The incremental cost-effectiveness ratio will be calculated and the cost-effectiveness acceptability curve will be graphed to summarise the uncertainty of cost-effectiveness according to different willingness to pay.

### Monitoring and reporting of risks

Standard Operational Procedures (SOPs) will be developed for risk management and reporting based on those used in previous clinical trials [15]. The SOPs will detail role responsibilities and processes for risk reporting and be reviewed and approved by a Data Safety Monitoring Board (DSMB) and trial Steering Committee. The DSMB members consist of an Obstetrician, midwife, statistician, and a consumer representative and provide independent oversight of the trial. The Steering Committee will consist of the Chief Investigators and Trial Manager and convene every 2 weeks. Each SOP will define and stratify any potential risk according to the University of Queensland Risk Level Calculator, which assesses the ‘likelihood’ versus the ‘consequences’ of risk and thereby assigns ratings from 1 = low to 5 = very high. The SOPs will also outline the standardised response and reporting procedure per risk level. Emergency code breaks for trial allocation will be available 24/7 if required. All adverse events will be reported and actioned immediately. The DSMB will manage the Risk Register, and recommend necessary modifications or termination of the trial. The DSMB will meet quarterly, or in response to serious adverse events and may undertake audits of trial conduct. Code breaking (unblinding) is possible in the event of a serious unexpected adverse event that requires identification of the allocated intervention to progress treatment. A sealed envelope containing the allocation will be held onsite in a secure location and accessible 24 h a day. Annual reports on trial progress will be provided to the Human Research Ethics Committee (HREC). The HREC will also be notified of any serious adverse events.

### Dissemination of findings

The results will be reported in conferences or peer-reviewed journals. The results will also be shared with participants, healthcare professionals and the public through lectures or science handbooks.

## Discussion

The provision of effective pain relief is a fundamental aspect of respectful, safe care during labour and birth [33]. Ideally, pharmacological analgesic options should support, rather than limit, women’s choices and plans to manage the pain of labour and be free of side effects that may negatively contribute to the experience and outcomes of labour. Currently none of the commonly available pharmacological options achieves this. What is needed is an analgesic option that is simple to administer, effective in reducing contraction pain, largely free of side effects and compatible with non-pharmacological techniques. The use of sterile water injections has the potential to fulfil this need. The technical simplicity of sterile water injections makes it suitable for use in all maternity care settings and by many levels of health care providers.

Our study will be the first placebo-controlled randomised trial to assess the use for sterile water injections to relieve the abdominal contraction pain of labour. The study design has the methodological strength to provide high level evidence efficacy and safety. The trial will also initiate the use of a vapocoolant spray to mitigate the pain associated with the administration of water injections and assess the impact of this approach on the acceptability of the procedure. The postnatal survey will assess women’s satisfaction with the allocated treatment and likelihood to reuse the same method in subsequent pregnancies. This experience of using water injections for abdominal labour pain will be further explored in proposed qualitative studies. The economic analysis will assess patterns and levels of resource utilisation associated with each participant across the two arms of the study. The combination of data and analysis will assist in contextualising findings from the RCT and enhance the understanding of the potential role of water injections as a labour pain analgesic.

The successful completion of our trial will see a new use for one of the most widely available medical preparations as an analgesic, improved choice for women and more efficient use of health resources.

## Trial status

Trial protocol version and date: Version 3 22 November 2021

Recruitment will commence 25th April 2022

Recruitment to be completed by 31st May 2023

## Supplementary Information

**Additional file 1.**

**Additional file 2.**

## Data Availability

Access to the full trial protocol and data from this study will be confidential until the trial is completed and the database is closed at the end of the study. Following final publications the protocol and database will be open to other researchers upon request in line with National Health and Medical Research Council policy.

## Abbreviations

GP: General Practitioner
MGP: Midwifery Group Practice
VAS: Visual analogue scale
SOP: Standard Operational Procedure
DSMB: Data Safety Monitoring Board
GCP: Good Clinical Practice
