# Correction: Sterile water injections for relief of labour pain (the SATURN trial): study protocol for a randomised controlled trial

**DOI:** 10.1186/s13063-022-06171-6

**Published:** 2022-04-15

**Authors:** Nigel Lee, Yu Gao, Lena B. Mårtensson, Leonie Callaway, Belinda Barnett, Sue Kildea

**Affiliations:** 1 School of Nursing Midwifery and Social Work, University of Queensland, Level 3 Chamberlain Building, St Lucia, Queensland Australia; 2 Molly Wardaguga Research Centre, College of Nursing & Midwifery, Charles Darwin University, Level 11, East Building, 410 Ann St., Brisbane, Queensland 4000 Australia; 3 School of Health Sciences, University of Skovde, Box 408, SE 54128 Skövde, Sweden; 4 Women’s and Newborn Services, Royal Brisbane and Women’s Hospital, Level 6, Ned Hanlon Building, Butterfield Street, Herston, Queensland 4029 Australia

**Correction to: Trials 23, 155 (2022)**

**https://doi.org/10.1186/s13063-022-06093-3**

The article [1] has been updated to include the changes made during peer review. The original version was published prematurely due to an error on the publisher’s side.
